# Time series analysis: trend in late maternal mortality in Brazil, 2010-2019

**DOI:** 10.1590/0102-311XEN168223

**Published:** 2024-08-26

**Authors:** Kelly Cristina Almeida Borgonove, Sônia Lansky, Vânia Muniz Nequer Soares, Fernanda Penido Matozinhos, Eunice Francisca Martins, Roberto Allan Ribeiro Silva, Kleyde Ventura de Souza

**Affiliations:** 1 Hospital das Clínicas, Universidade Federal de Minas Gerais, Belo Horizonte, Brasil.; 2 Secretaria Municipal de Saúde Belo Horizonte, Belo Horizonte, Brasil.; 3 Associação Brasileira de Obstetrizes e Enfermeiros Obstetras, Rio de Janeiro, Brasil.; 4 Escola de Enfermagem, Universidade Federal de Minas Gerais, Belo Horizonte, Brasil.; 5 Universidade Federal dos Vales do Jequitinhonha e Mucuri, Janaúba, Brasil.

**Keywords:** Maternal Mortality, Maternal Death, Postpartum Period, Cause of Death, Health Information Systems, Mortalidad Materna, Muerte Materna, Periodo Posparto, Causas de Muerte, Sistemas de Información em Salud

## Abstract

To analyze the temporal trend of the late maternal mortality ratio (LMMR) in Brazil and its geographic regions in the period from 2010 to 2019, an ecological time series study was conducted. Data related to late maternal mortality from information systems of the Brazilian Ministry of Health were used. Statistical analysis used Prais-Winsten autoregressive models. A total of 1,470 late maternal deaths were reported in Brazil, resulting in an LMMR of 5 deaths per 100,000 live births. The late maternal mortality records revealed regional disparities, with the lowest index in the North (3.5/100,000 live births) and the highest in the South (8.3/100,000 live births). The LMMR showed an increasing trend in the country, with a general increase in the LMMR in the period and a mean annual percentage variation of 9.79% (95%CI: 4.32; 15.54). The Central-West region led this increase, with a mean annual percentage change of 26.06% (95%CI: 16.36; 36.56), followed by the North and Northeast regions, with 23.5% (95%CI: 13.93; 33.88). About 83% of the reported late maternal deaths were investigated, and 65.6% were corrected by the Maternal Mortality Committees. These findings highlight the relevance of late maternal mortality as an important indicator for maternal health, which is often invisible. The increase in the LMMR result from the improvement in the quality of the registration of these deaths in recent years in Brazil, and especially from the work of investigating deaths. The fragility of reporting with regional disparities points to the need for a more comprehensive approach that promotes equity and prevention of avoidable late maternal mortality.

## Resumen

Con el objetivo de evaluar la tendencia temporal de la tasa de mortalidad materna tardía (TMMT) en Brasil y sus regiones geográficas para el período de 2010 a 2019, se realizó un estudio de serie temporal ecológica. Se utilizaron datos relacionados con la mortalidad materna tardía de los sistemas de información del Ministerio de la Salud de Brasil. El análisis estadístico empleó modelos de regresión de Prais-Winsten. Hubo 1.470 muertes maternas tardías en Brasil, lo que resultó en una TMMT de 5 muertes por cada 100.000 nacidos vivos. Los registros de mortalidad materna tardía revelaron disparidades regionales con la tasa más baja en la Región Norte (3,5/100.0000 nacidos vivos) y la más alta en la Región Sur (8,3/100.000 nacidos vivos). Hubo una tendencia a aumento de TMMT en el país, con un incremento general de TMMT para el período y una variación media porcentual anual de un 9,79% (IC95%: 4,32; 15,54). La Región Centro-oeste presentó las tasas más elevadas, con una variación media porcentual anual de un 26,06% (IC95%: 16,6; 36,56), seguida de las regiones Norte y Nordeste, con un 23,5% (IC95%: 13,93; 33,88). Aproximadamente el 83% de las muertes materna tardía reportadas fueron investigadas, y el 65,6% fue corregido por los Comités de Mortalidad Materna. Estos hallazgos muestran la relevancia de la mortalidad materna tardía como un indicador de importancia para la salud materna, muchas veces invisibilizada. El incremento en la TMMT encontrada puede deberse a la mejora en la calidad del registro de estas muertes en los últimos años en Brasil, especialmente de la investigación de las muertes. La debilidad de las notificaciones con disparidades regionales apunta a la necesidad de un enfoque más integral que promueva la equidad y la prevención de la mortalidad materna tardía evitable.

## Introduction

Maternal mortality is defined as the death of women from direct or indirect obstetric causes during pregnancy or up to 42 days after delivery, regardless of the duration or location of pregnancy, except for external causes of death ^1^. From 43 days after delivery to one year after the end of pregnancy, death is classified as late maternal mortality ^2^.

Preventable in 92% of cases, maternal deaths reflect the lack of coordination, disorganization, and unsatisfactory quality of health care, as well as their relationship with socioeconomic, racial/ethnic, and cultural factors ^3^. It is, therefore, an important indicator of social inequalities in access, coverage, and quality of health care, often associated with a sequence of events that includes inadequate interventions, omissions, and incorrect and inappropriate treatments, reflecting a mismatch with the health needs of women and the general population ^2^ ^,^ ^3^ ^,^ ^4^.

Aggravated by structural and institutional racism, maternal deaths reflect the intersectionality of social, gender, and ethnic-racial inequalities, with a disproportionate impact on Black and Indigenous women for decades, highlighting historical gaps in social justice and reproductive justice ^5^.

The maternal mortality ratio (MMR), calculated by the number of deaths of women from causes related to pregnancy, childbirth and puerperium (up to 42 days after delivery) per 100,000 live births is used to assess the living conditions of a population. Due to its relevance, it is part of the Sustainable Development Goals (SDGs), which have among their goals to reduce the global MMR of maternal mortality to less than 70 per 100,000 live births. In Brazil, this target was adapted to make it more accurate to the country’s context and challenges, being set for a maximum of 30 deaths per 100,000 live births ^6^.

Despite the decrease in MMR worldwide, from 385 to 216 per 100,000 live births from 1990 to 2015, maternal death is still a global public health concern ^3^. From 2016 to 2020, MMR reduced significantly in 31 countries in the Americas, stagnated in 133 countries, and increased significantly in 17 countries, seven of which were located in Latin America and the Caribbean, including Brazil ^7^.

Data from the Global Burden of Disease ^8^ showed that, in Brazil, MMR reduced from 111.4 to 62.1/100,000 live births from 1990 to 2019, but the country did not reach the Millennium Development Goals (MDG) of reducing MMR by 75% by 2015. The authors point out that the sharpest decline in MMR occurred between 1990 and 2000, with a decrease of 43%, and remained stable with oscillation over the following period. The MMR trends were marked by marked regional disparities in 2019, with variations ranging from 38.3 to 82.5/100,000 live births in the South and North regions, respectively ^3^.

The COVID-19 pandemic aggravated this reality, negatively impacting the projections of maternal mortality indicators, regressing to the level of decades ago, an increase in the number of maternal deaths of almost 100%, from 55.31 per 100,000 live births in 2019 to 107.53 per 100,000 live births in 2021 ^7^ and changed the composition of causes of death. This becomes evident when deaths from COVID-19 - infectious causes - surpassed other causes ^9^.

The death of women during the pregnancy-puerperal cycle persists as a serious public health problem, requiring a broad effort to cope with it. However, even in the face of political, social, and health strategies and actions, women continue to have their right to life threatened and die during the pregnancy, childbirth, and postpartum periods, often from avoidable causes ^10^ ^,^ ^11^ ^,^ ^12^ ^,^ ^13^, revealing a disturbing reality due to its magnitude, underreporting, and preventability. We add to these the deaths that make up the late maternal mortality ratio (LMMR), which occur in the late puerperium, constituting a little-explored aspect of this complex issue ^4^ ^,^ ^11^ ^,^ ^14^ ^,^ ^15^.

The study conducted by Cosio et al. ^14^ investigated the trend of late maternal mortality in seven countries in the Americas, selected due to their high maternal mortality, as well as the quality and availability of data on late maternal deaths and deaths due to obstetric sequelae in the period from 1999 to 2013. In these countries, including Brazil, an upward late maternal mortality trend has been identified, with the exception of Canada and Cuba. Comparing the periods from 1999 to 2005 and from 2006 to 2013, the group of countries analyzed showed a percentage variation of growth in late maternal mortality of 145.8%, whereas in Brazil this variation was 84.7%.

A study carried out in one of the capitals of northeastern Brazil sought to evaluate the contribution of the Maternal Mortality Committee in the qualification of the causes of death of women of childbearing age and late maternal mortality from 2010 to 2017. The results show that the lack of adequate identification of late maternal deaths reflects the persistent invisibility of this serious and important event, both from a social and health point of view ^15^.

In view of the scarcity of studies on late maternal mortality, whose information is essential to support public policies, actions, and coping strategies, the theme raises concerns and questions due to the extent of the problem ^14^ ^,^ ^16^. This study aimed to analyze the temporal trend of the late maternal mortality ratio (LMMR) in Brazil and its geographic regions in the period from 2010 to 2019, using data from the Brazilian Mortality Information System (SIM) and the Brazilian Information System on Live Birth (SINASC) of the Brazilian Ministry of Health.

## Methodology

This is a study with an exploratory ecological design of time series, using late maternal mortality data from Brazil and geographic regions, in the interval from 2010 to 2019. The data come from SIM and SINASC, available on the Health Portal of the Brazilian Informatics Department (DATASUS) of the Brazilian Ministry of Health. These systems provide the basis for generating epidemiological indicators, functioning as a strategic matrix, and can support decision-making in various areas of health care ^17^.

The data were accessed by using DATASUS (https://datasus.saude.gov.br/). The information contained in the Death Certificates (DC) was obtained from the “Services” directory (in the File Transfer/Download section of SIM/DATASUS/MS). Data on live births were collected in the “Health Information” directory (TABNET), in the area of Vital Statistics, option Live Births - 1994 to 2019. To use data on maternal race/color of live births, the files available in the “Services” directory were imported (in the File Transfer/Download section of SINASC/DATASUS/MS).

To read/convert the files, the statistical analysis application TabWin (http://siab.datasus.gov.br/DATASUS/index.php?area=060805&item=3), an open access tool developed by DATASUS, was used. The databases were organized in Microsoft Excel (https://products.office.com/) spreadsheets and later filtered for analysis of the selected cases with the underlying cause of death recorded with International Classification of Diseases, 10th revision (ICD-10) code O96, indicating a late maternal death.

Thus, the population of this study consisted of all deaths identified as late maternal mortality from the registration of ICD-10 code O96 on the DC, registered in SIM, of residents in the Brazilian territory, in the period from January 1, 2010, to December 31, 2019. The choice of this investigation period was due to the modifications in the previous versions of the DC, to ensure greater reliability in the information.

Regarding the descriptive data, the analysis and presentation of tables and graphs were performed using Microsoft Excel software. The variables were expressed as absolute and relative frequencies. The LMMR was calculated using the formula [(number of late maternal deaths/number of live births in the same period) x 100,000]. Although important to reduce inaccuracies in the records that may lead to underreporting of maternal deaths ^18^, no correction factor was applied to the observed values, due to the lack of proposals for national correction factors indicated for late maternal mortality ^19^.

The Brazilian Ministry of Health recommends the use of a correction factor for maternal deaths for the purpose of calculating MMR when the increase in the number of maternal deaths in SIM after the death surveillance process is less than 34.3% (gold standard). However, note the lack of a reference on validating the use of correction factors for late maternal mortality ^19^.

The categorization of sociodemographic variables and other factors associated with late maternal mortality followed the structure of the SIM ^17^. Regarding the variables age group and race/color, the categorization of age groups used by the Brazilian Institute of Geography and Statistics (IBGE) in the *2022 Demographic Census* ^20^ was adopted.

The statistical package Statistical Software for Professional (Stata, https://www.stata.com), version 16.0, was used to analyze the trends. Considering the nonstationary nature of the time series of maternal mortality, Prais-Winsten autoregressive models were used, in which the dependent variables were the late maternal mortality ratios for Brazil and the five geographic regions of the country.

The Prais-Winsten regression model was adopted for being indicated to correct serial autocorrelation from time series. To perform the regression, the LMMR were transformed to the logarithmic scale. This process is performed to reduce the heterogeneity of the variance of the residuals from the time series regression analysis ^18^ ^,^ ^19^.

In addition, the annual mean percentage change (APC) was calculated for each dependent variable analyzed. The formula used to calculate the APC was as follows: APC = (-1+10[b1]*100%), where b1 represents the slope coefficient (beta) of the Prais-Winsten regression ^18^ ^,^ ^19^. The 95% confidence intervals (95%CI) of the APC measurements were calculated according to the formulas minimum 95%CI (-1+10[b1-te]*100%) and maximum 95%CI (-1+10[b1+te]*100%).

The values of the slopes (b1) of the Prais-Winsten regression and standard errors were generated by the statistical analysis program; Student’s t-test was performed with 9 degrees of freedom (t = 2.262 for the 10-year period) and with 7 degrees of freedom (t = 2.365 for the 8-year period) for the maternal race/color variable, both with a 95% confidence level.

The regression results were interpreted as follows: increasing trend when the p-value was less than 0.05 and the regression coefficient was positive; a decreasing trend when the p-value was less than 0.05 and the regression coefficient was negative; or stationary trend when the p-value was greater than 0.05 ^21^.

This study used public data, available to researchers and citizens, without restrictions. Such information was obtained from sources that presented it in an aggregated form, ensuring the anonymity of the women. Therefore, according to *Resolution n. 510/2016* of the Brazilian National Health Council, it was not necessary to submit the study to a Human Research Ethics Committee.

## Results

In the period between 2010 and 2019, 1,470 late maternal deaths were reported in Brazil, with an annual average of 147 deaths (SD = 113-181). In 2018, the highest number of deaths was recorded, totaling 209 cases, corresponding to 14.2% of all late maternal deaths in the period. On the other hand, 2010 showed the lowest number of records, with 63 late maternal deaths declared, equivalent to 4.3% of the total number of deaths.

Late maternal deaths in Brazil predominated among women over 45 years of age, those with low schooling, and those registered as widows. Regarding race/skin color, 59.1% (n = 869) occurred in black women over the ten years investigated, with an LMMR of 5.7 per 100,000 live births, equal to the LMMR of white women, who accounted for 36.9% (n = 542) of the total deaths.

Of the late maternal deaths reported in Brazil during the study period, 1,230 cases were investigated, corresponding to 83.7% of the total. A total of 131 cases were not investigated (8.9%), whereas 109 cases (7.4%) lacked a response to this variable. Among the deaths investigated, 65.6% of the late maternal mortality cases lacked the underlying cause record with the code for late maternal death on the original DC, and these data were corrected after investigation by the Maternal Mortality Committees.

The Southeast Region stood out for the highest percentage of investigation of late maternal mortality cases, with 92.5% (n = 483) of late maternal deaths investigated between 2010 and 2019. The lowest percentages of investigation were observed in the Northeast Region with 53.3% (n = 218), followed by the South Region with 77.3% (n = 249), and the Central-West and North regions showed similar coverage, 84.1% (n = 90) and 84.5% (n = 93), respectively.

At the end of the ten years investigated, the Southeast Region had the highest number of late maternal mortality cases, with 522 deaths recorded (35.5%), whereas the Central-West Region had the lowest number, declaring 107 deaths in the same period (7.3%). Note that the North Region did not report late maternal deaths in 2010 and 2011.

The evaluation of the Federative Units (UF, acronym in Portuguese) revealed that Rio de Janeiro State registered the highest number of deaths, 280 cases (19%), followed by Rio Grande do Sul State, with 213 deaths registered (14.5%). The Federal District did not report any late maternal deaths throughout the decade, whereas Acre State and Amapá State recorded only one death in the period. Table 1 shows the distribution of late maternal deaths by Brazilian UF/Geographic Regions between 2010 and 2019.

**Table 1:** Late maternal deaths. Brazil, regions and Federative Units (UF, acronym in Portuguese), 2010-2019.

Geographic regions/UF	2010-2011		2012-2013		2014-2015		2016-2017		2018-2019		2010-2019	
	n	%	n	%	n	%	n	%	n	%	n	%
Central-West	8	4.5	13	5.4	14	3.9	36	11.0	36	9.8	107	7.3
Federal District	-	-	-	-	-	-	-	-	-	-	-	-
Goiás	-	-	1	-	-	-	5	-	11	-	17	1.2
Mato Grosso	2	-	2	-	7	-	17	-	20	-	48	3.3
Mato Grosso do Sul	6	-	10	-	7	-	14	-	5	-	42	2.9
Northeast	47	26.6	56	23.2	91	25.5	77	23.6	138	37.4	409	27.8
Alagoas	4	-	-	-	-	-	1	-	-	-	5	0.3
Bahia	6	-	21	-	20	-	19	-	13	-	79	5.4
Ceará	9	-	8	-	19	-	6	-	57	-	99	6.7
Maranhão	9	-	6	-	7	-	2	-	1	-	25	1.7
Paraíba	-	-	7	-	8	-	10	-	17	-	42	2.9
Pernambuco	1	-	-	-	18	-	22	-	30	-	71	4.8
Piauí	16	-	11	-	16	-	13	-	15	-	71	4.8
Rio Grande do Norte	1	-	-	-	-	-	1	-	4	-	6	0.4
Sergipe	1	-	3	-	3	-	3	-	1	-	11	0.7
North	-	-	14	5.8	23	6.4	35	10.7	38	10.3	110	7.5
Acre	-	-	1	-	-	-	-	-	-	-	1	0.1
Amapá	-	-	-	-	1	-	-	-	-	-	1	0.1
Amazonas	-	-	3	-	4	-	12	-	19	-	38	2.6
Pará	-	-	5	-	10	-	11	-	13	-	39	2.6
Rondônia	-	-	4	-	6	-	6	-	4	-	20	1.4
Roraima	-	-	-	-	-	-	1	-	1	-	2	0.1
Tocantins	-	-	1	-	2	-	5	-	1	-	9	0.6
Southeast	65	36.7	94	39.0	136	38.1	118	36.2	109	29.5	522	35.5
Espírito Santo	5	-	14	-	29	-	21	-	13	-	82	5.6
Minas Gerais	1	-	4	-	13	-	10	-	7	-	35	2.4
Rio de Janeiro	46	-	52	-	54	-	60	-	68	-	280	19
São Paulo	13	-	24	-	40	-	27	-	21	-	125	8.5
South	57	32.2	64	26.6	93	26.1	60	18.4	48	13.0	322	21.9
Paraná	19	-	12	-	29	-	18	-	26	-	104	7.1
Rio Grande do Sul	38	-	52	-	62	-	41	-	20	-	213	14.5
Santa Catarina	-	-	-	-	2	-	1	-	2	-	5	0.3
Brazil	177	100.0	241	100.0	357	100.0	326	100.0	369	100.0	1,470	100.0

Source: prepared by the author with data from Brazilian Health Informatics Department ^17^.

Regarding the distribution of late maternal deaths by municipality during the study period, the city of Rio de Janeiro stands out, with 175 cases, corresponding to 11.9% of all deaths reported in the country. In Porto Alegre (Rio Grande do Sul State), the second municipality with the highest number of cases, 45 late maternal deaths (3.1%) were declared in the ten years of the investigation.

In the analysis of late maternal mortality in the five geographic regions of the country throughout the period studied, the North Region had the lowest LMMR, 3.5 per 100,000 live births, and the the South Region had the highest, 8.3 per 100,000 live births, followed by the Northeast Region, where the LMMR was 4.9/100,000 live births. At the end of the ten years, the Central-West and Southeast regions had the same LMMR, with 4.5 per 100,000 live births.

**Figure 1:**
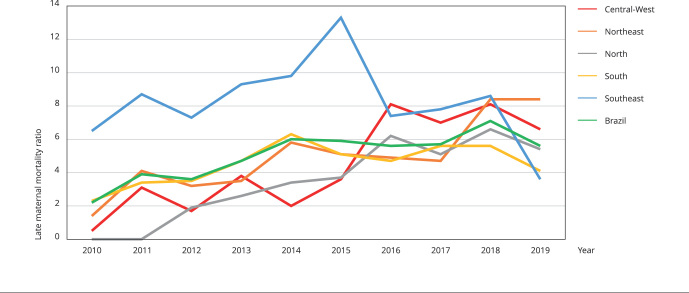
Late maternal mortality ratio. Brazil and geographic regions, 2010-2019.

Figure 1 shows the behavior of the LMMR for Brazil and its regions over the years studied. At the beginning of the period, the LMMR for Brazil was 2.2 deaths per 100,000 live births, evolving to 5.6 deaths per 100,000 live births in 2019. In the decade studied, the LMMR in Brazil was 5 deaths per 100,000 live births. Variation in the LMMR was observed in the five geographic regions of the country: in the initial triennium (2010-2012) the lowest LMMR were found in the Central-West, Northeast, and North regions, with a progressive increase, surpassing the other regions at the end of the period studied.

The analysis of the Prais-Winsten regression coefficients for Brazil and for each of the five geographic regions showed a significant increasing trend of the LMMR in Brazil (p = 0.003) and in the Central-West, Northeast and North regions (p < 0.001 in the three regions), and stationary trend in the South and Southeast regions.

The mean annual percentage variations in Brazil and in the five geographic regions showed an increase in the LMMR in Brazil, with a mean annual percentage variation of 9.79% (95%CI: 4.32; 15.54) (Table 2). In the analysis according to region, the Central-West showed the highest increase in the LMMR, with a mean annual percentage change of 26.06% (95%CI: 16.36; 36.56), followed by the North Region, with 23.5% (95%CI: 13.93; 33.88), and the Northeast Region, with 14.6% (95%CI: 8.50; 21.05). The other regions showed a stationary trend.

**Table 2:** Mean percentage change in the late maternal mortality ratio. Brazil and regions, 2010-2019.

	% mean annual change	95%CI	p-value	tendency
Brazil	9.79	4.32; 15.54	0.003	Increasing
Geographic regions				
Central-West	26.06	16.36; 36.56	< 0.001	Increasing
Northeast	14.60	8.50; 21.05	< 0.001	Increasing
North	23.50	13.93; 33.88	< 0.001	Increasing
Southeast	6.74	-1.19; 15.30	0.092	Stationary
South	-3.26	-11.20; 5.39	0.407	Stationary

95%CI: 95% confidence interval.

Source: prepared by the author with data from Brazilian Health Informatics Department ^17^.

At the end of the study period, we found that 77.3% of the women who died in the late puerperium (n = 1,137) received medical care during the illness that caused the death. The five-year analysis revealed an increase in health care coverage, with coverage of 70% (n = 417) between 2010-2014 and 82.4% (n = 720) between 2015-2019. Note the significant percentage of cases in which this information was not available, notably between 2010 and 2014, when this data was not recorded in 22.3% of the occurrences (n = 133). However, the quality of information improved in the second five-year period, with 9.6% of the occurrences (n = 84) lacking the recording of this data.

## Discussion

The findings of this study show a growing trend of LMMR in Brazil and in most of its regions over the decade from 2010 to 2019, with a predominance of these deaths among women over 45 years of age, black, with low schooling, and widows. These data indicate the historical gaps that show the social and reproductive injustice that marks women’s lives ^5^ ^,^ ^22^. In addition, the notification had important flaws and the information in the original DC was incomplete.

Previous studies investigating deaths in the Americas from 1999 to 2013, such as the study conducted by Cosio et al. ^14^, suggest that the upward trend in late maternal mortality can be seen in Brazil since at least the 1990s. Carvalho et al. ^15^, point out that, in developed countries, the increase in late maternal mortality is associated with increased survival, but can also be attributed to the expansion of the investigation of women of childbearing age deaths over the years with the correction of underreporting, active search and qualification, and the use of information systems.

Note that in Brazil, the regulation of the investigation of maternal mortality by municipalities, throughout the national territory, was established by *Ordinance n. 1,119* of the Brazilian Ministry of Health only in 2008 ^3^. The organization of municipal surveillance for this purpose, despite previous initiatives with the implementation of Maternal Mortality Committees in some states since the 1990s, only took place from 2009 onwards, which partly explains the increase in the identification and notification of maternal deaths in the years that followed.

Despite the global decrease in late maternal mortality, from 8,460 (95%CI: 5,792; 11,935) in 1990 to 6,711 (95%CI: 4,335; 9,996) in 2015 ^8^, several international studies and reports ^8^ ^,^ ^14^ ^,^ ^23^ ^,^ ^24^ ^,^ ^25^ ^,^ ^26^ ^,^ ^27^ ^,^ ^28^ ^,^ ^29^ ^,^ ^30^ indicate that late maternal mortality is not only a challenge in Brazil, affecting countries of different levels of development. In the Americas, between 1999 and 2013, an increasing trend in late maternal mortality was observed, from 1,179 deaths between 1999 and 2006 to 3,153 between 2006 and 2013, with a mean annual percentage change of 12.4, with the largest difference observed in the United States (15.4%), followed by Mexico (15.1%) ^14^. The LMMR and MMR for obstetric sequelae was 2 times higher in the Americas region between 2006 and 2013, compared with the period from 1999 to 2005.

This study verified an LMMR of 5 deaths per 100,000 live births between 2010 and 2019 for Brazil. Official reports indicate an LMMR of 13.7 deaths per 100,000 live births between 2018 and 2020 in the United Kingdom ^25^ and 2.7 deaths per 100,000 live births between 2013 and 2015 in France ^24^. Part of the discrepancy in rates between countries is due to differences in registration and quality of information.

In Brazil, although the Ministry of Health has sought to develop estimates of MMR that can be reliably applied at the subnational level, considering regional inequalities, these estimates do not include late maternal mortality due to the low completeness and reliability of the investigation of these deaths. This is aggravated by the scarce production of knowledge on this subject. The limitations in the identification and recording of late maternal mortality result from the temporal distance between the occurrence of death and delivery, making it difficult to define the causal link between the cause of death and the conditions of pregnancy, childbirth, and/or puerperium ^31^.

This difficulty may also be related to the underreporting or scarcity of information of these deaths, which requires a review and greater detailing of the ICD-10 codes of late maternal deaths, as well as extensive training of healthcare providers, especially physicians, professionals responsible for filling out the DC, as well as death coding technicians in Brazil and professionals who make up maternal death surveillance committees ^13^ ^,^ ^31^. Underreporting is also identified in developed countries ^8^ ^,^ ^23^ ^,^ ^24^.

In addition, the correct assignment of ICD-10 codes for maternal and late maternal deaths is difficult in the process of coding death certificates. This challenge mainly affects areas of lower socioeconomic status, where maternal mortality tends to be higher ^31^ ^,^ ^32^.

The use of codes referring to late maternal deaths (O96) in the ICD-10, included by the WHO in the revision of ICD-10 in 2015 (codes O96.0 - direct obstetric late maternal death; O96.1 - indirect obstetric maternal death; and O96.9 - for undefined causes), should be implemented, considering the magnitude of late maternal mortality, with the training of epidemiological surveillance teams, committees, and coders, with a view to improving surveillance, investigation, coding, and analysis of late maternal deaths. Adopting this codification in Brazil will offer greater reliability and consequent visibility to late maternal deaths ^1^.

Authors highlight he complexity of measuring late maternal mortality due to underreporting and the challenges in diagnosing the underlying causes of death ^13^. Although the notification of maternal death is mandatory, underreporting hinders the actual dimensioning of these deaths ^4^ ^,^ ^8^ ^,^ ^16^ ^,^ ^13^ ^,^ ^33^. In Brazil, even in the face of considerable advances in official death records, with investigation reaching a coverage of more than 90%, this coverage is not uniform throughout the country, ranging from 53.3% in the Northeast to 92.3% in the Southeast.

The Maternal Mortality Committees play a decisive role in identifying and correcting the underreporting of maternal deaths, especially late deaths, whose causes are complex to identify and analyze. Vega et al. ^13^ emphasize that without the work of the committees, late maternal deaths would remain hidden, given their greater underreporting compared with deaths up to 42 days of the puerperium.

Note that the late maternal mortality scenario in Brazil differs from that of MMR (up to 42 days). While the LMMR shows an increasing trend from 2010 to 2019, the MMR showed a trend of stagnation, with a slight decrease until the pandemic period, when there was a sharp increase, almost doubling the MMR in the country ^34^. Both indicators present regional disparities, indicating that, in the same period of the study, the MMR lowered significantly in the Central-West, Northeast, and South regions ^35^. On the other hand, an increasing trend of LMMR was evidenced in the Central-West, Northeast, and North regions, whereas a trend of stability was identified in the Southeast and South regions.

This stationary trend of MMR can be partly explained by the so-called perinatal paradox, which highlights the persistence of high maternal mortality rates, despite technological advances and available resources. This is attributed to the hyper-medicalization of the labor and birth process, characterized by the excess of interventions that impact the natural physiological course, resulting in an increased risk of complications and mortality ^36^.

The increasing LMMR in the country may suggest an improvement in the quality of the information on these deaths, with an increase and qualification of the investigation of maternal death over the period. On the other hand, it may indicate inequality in maternal survival due to problems and disparities in access, availability, and quality of health services, infrastructure, education, and socioeconomic conditions, such as access to safe prenatal care, childbirth, and postpartum follow-up in different regions of Brazil ^34^. Better understanding regional differences is essential, to guide the development of public health policies in the country according to local needs.

Recognizing the importance of the LMMR and its inclusion in health indicators, in government commitments, and in health planning is essential for the understanding and effective approach and prevention of maternal deaths. Although the 2030 Agenda of the SDGs only aims to reduce maternal mortality up to 42 days of puerperium, including the reduction of late maternal mortality would be advisable in future development goals and objectives of Brazil and all countries, considering the evidence of its magnitude, transcendence, and avoidability ^4^ ^,^ ^14^ ^,^ ^15^. In addition, the most recent official statistics from the Brazilian Ministry of Health show that Brazil is considerably far from meeting the current SDG targets ^35^.

This study has limitations, including the use of secondary data from death records and the high proportion of underreporting. The results, therefore, should be understood as restricted to the subset of deaths registered in the SIM, in view of the possibility of information bias, especially in regions where underreporting of maternal deaths is higher. However, the analyses carried out in this study show for the first time an overview of late maternal mortality in Brazil for a decade, 2010-2019, with the potential to foster discussions and guide advances in health actions that reach all women. The invisibility of late maternal deaths, evidenced by their underreporting in official information systems, and also revealed in this study, constitutes another aspect of reproductive injustice in our country, deepening Brazil’s gap and delay regarding health, the guarantee of reproductive rights and of a dignified life for women.

In particular, the importance of focusing special attention on the follow-up of women during the late puerperium, considering the potential positive impact on the prevention of these avoidable deaths, stands out. Therefore, future research should incorporate an in-depth analysis of the underlying causes of late maternal mortality, as well as interventions in professional training and in improving the quality of health services

These and other measures can contribute to achieving the commitment to reduce maternal mortality and promote women’s health, highlighting the challenges associated with the care and care of women in the puerperium.
