# Supplementary material for: Time series analysis: trend in late maternal mortality in Brazil, 2010-2019
Source: Cad Saude Publica. 2024 Aug 26;40(7):e00168223. doi: 10.1590/0102-311XEN168223 (PMC11349278; doi:10.1590/0102-311XEN168223)
Supplement: Translation

# Translation: Análise de série temporal: tendência da mortalidade materna tardia no Brasil, 2010-2019

**DOI:** 10.1590/0102-311XPT168223

**Authors:** Kelly Cristina Almeida Borgonove, Sônia Lansky, Vânia Muniz Nequer Soares, Fernanda Penido Matozinhos, Eunice Francisca Martins, Roberto Allan Ribeiro Silva, Kleyde Ventura de Souza

**Affiliations:** 1 Hospital das Clínicas, Universidade Federal de Minas Gerais, Belo Horizonte, Brasil.; 2 Secretaria Municipal de Saúde Belo Horizonte, Belo Horizonte, Brasil.; 3 Associação Brasileira de Obstetrizes e Enfermeiros Obstetras, Rio de Janeiro, Brasil.; 4 Escola de Enfermagem, Universidade Federal de Minas Gerais, Belo Horizonte, Brasil.; 5 Universidade Federal dos Vales do Jequitinhonha e Mucuri, Janaúba, Brasil.

**Keywords:** Mortalidade Materna, Morte Materna, Período Pós-Parto, Causas de Morte, Sistemas de Informação em Saúde

## Abstract

Com o propósito de analisar a tendência temporal da razão de mortalidade materna tardia (RMMT) no Brasil e suas regiões geográficas no período de 2010 a 2019, conduziu-se um estudo ecológico de série temporal. Foram utilizados dados relacionados à mortalidade materna tardia, provenientes de sistemas de informação do Ministério da Saúde. A análise estatística empregou modelos autorregressivos de Prais-Winsten. Foram notificados 1.470 óbitos maternos tardios no Brasil, resultando em uma RMMT de 5 óbitos a cada 100 mil nascidos vivos. Os registros de mortalidade materna tardia revelaram disparidades regionais com o menor índice na Região Norte (3,5/100 mil nascidos vivos) e o maior na Região Sul (8,3/100 mil nascidos vivos). Houve tendência crescente da RMMT no país, com aumento geral no período e variação percentual média anual de 9,79% (IC95%: 4,32; 15,54). A Região Centro-oeste liderou esse aumento, com variação percentual média anual de 26,06% (IC95%: 16,36; 36,56), seguida pelas regiões Norte e Nordeste, com 23,5% (IC95%: 13,93; 33,88). Cerca de 83% das mortes maternas tardias declaradas foram investigadas, sendo que 65,6% foram corrigidas pelos Comitês de Mortalidade Materna. Esses achados ressaltam a relevância da mortalidade materna tardia como um indicador de importância para a saúde materna muitas vezes invisibilizado. O aumento da RMMT verificado pode ser resultado da melhoria da qualidade do registro desses óbitos nos últimos anos no Brasil, sobretudo do trabalho de investigação dos óbitos. A fragilidade das notificações com as disparidades regionais aponta a necessidade de uma abordagem abrangente que promova equidade e prevenção de mortalidade materna tardia evitáveis.

## Introdução

A mortalidade materna é definida como o óbito de mulheres por causas obstétricas diretas ou indiretas durante a gestação ou até 42 dias após o parto, independentemente de duração e localização da gravidez, excetuando-se as causas externas de óbito ^1^. A partir de 43 dias após o parto, até um ano após o término da gestação, a morte é classificada como mortalidade materna tardia ^2^.

Evitável em 92% dos casos, a morte materna reflete falta de coordenação, desorganização e qualidade insatisfatória da assistência de saúde, e tem relação com fatores socioeconômicos, raciais/étnicos e culturais ^3^. É, portanto, um importante indicador de desigualdades sociais no acesso, cobertura e qualidade dos cuidados de saúde, frequentemente associado a uma sequência de eventos que inclui intervenções inadequadas, omissões e tratamentos incorretos e inapropriados, refletindo descompasso com as necessidades de saúde das mulheres e da população em geral ^2^ ^,^ ^3^ ^,^ ^4^.

Agravadas pelo racismo estrutural e institucional, as mortes maternas espelham a interseccionalidade das desigualdades sociais, de gênero e étnico-raciais, tendo impacto desproporcional sobre mulheres negras e indígenas há décadas, evidenciando lacunas históricas de justiça social e reprodutiva ^5^.

A razão de mortalidade materna (RMM), calculada pelo número de mortes de mulheres por causas relacionadas à gestação, parto ou puerpério (até 42 dias após o parto) por 100 mil nascidos vivos, é utilizada para avaliar a condição de vida de uma população. Por sua relevância, integra os Objetivos de Desenvolvimento Sustentável (ODS), que têm entre suas metas reduzir a RMM global para menos de 70 por cada 100 mil nascidos vivos. No Brasil, essa meta foi adaptada a fim de torná-la mais precisa aos contexto e desafios do país, sendo definida para no máximo 30 mortes por 100 mil nascidos vivos ^6^.

Apesar do registro de queda da RMM no mundo, de 385 para 216 por 100 mil nascidos vivos entre 1990 e 2015, a mortalidade materna persiste como preocupação para a saúde pública global ^3^. De 2016 a 2020 houve redução significativa da RMM em 31 países das Américas, estagnação em 133 países e aumento significativo em 17 países, sete dos quais localizados na América Latina e Caribe, incluindo o Brasil ^7^.

Dados do estudo *Carga Global de Doenças* ^8^ demonstraram que, no Brasil, houve redução da RMM de 111,4 para 62,1/100 mil nascidos vivos de 1990 a 2019, porém o país não atingiu o Objetivo de Desenvolvimento do Milênio (ODM) de reduzir 75% da RMM até 2015. Os autores ressaltam que o declínio mais acentuado na RMM ocorreu entre 1990 e 2000, com diminuição de 43%, e manteve-se estável, com oscilação ao longo do período seguinte. As tendências na RMM foram marcadas por acentuadas disparidades regionais em 2019, com variações que oscilaram de 38,3 a 82,5/100 mil nascidos vivos nas regiões Sul e Norte, respectivamente ^3^.

A pandemia de COVID-19 agravou essa realidade, impactando negativamente as projeções dos indicadores de mortalidade materna, retrocedendo ao patamar de décadas atrás, com incremento no número de mortes maternas de quase 100%, passando de 55,31 por 100 mil nascidos vivos em 2019 para 107,53 por 100 mil nascidos vivos em 2021 ^7^, com mudanças na composição das causas de óbito. Isso se evidencia com a ultrapassagem das demais causas pelas mortes por COVID-19 - causas infecciosas ^9^.

A morte de mulheres durante o ciclo gravídico-puerperal persiste como um grave problema de saúde pública, demandando amplo esforço para seu enfrentamento. Mesmo com estratégias e ações de cunho político-social e sanitário, as mulheres seguem com seu direito à vida ameaçado, morrendo nos períodos de gestação, parto e pós-parto, por causas frequentemente evitáveis ^10^ ^,^ ^11^ ^,^ ^12^ ^,^ ^13^, revelando uma realidade inquietante por sua magnitude, subinformação e evitabilidade. Somam-se a esses os óbitos que compõem a razão de mortalidade materna tardia (RMMT), que ocorrem no puerpério tardio, face pouco explorada dessa complexa questão ^4^ ^,^ ^11^ ^,^ ^14^ ^,^ ^15^.

O estudo realizado por Cosio et al. ^14^ investigou a tendência da mortalidade materna tardia em sete países das Américas, incluindo o Brasil, selecionados devido à alta mortalidade materna, bem como pela qualidade e disponibilidade de dados sobre mortes maternas tardias e mortes por sequelas obstétricas no período de 1999 a 2013. Nesses países foi identificada uma tendência ascendente na mortalidade materna tardia, com exceção de Canadá e Cuba. Comparando-se os períodos de 1999 a 2005 e de 2006 a 2013, o grupo de países analisados apresentou variação percentual de crescimento na mortalidade materna tardia de 145,8%, enquanto no Brasil essa variação foi de 84,7%.

Um estudo realizado em uma das capitais do nordeste brasileiro buscou avaliar a contribuição do Comitê de Mortalidade Materna na qualificação das causas de óbito de mulheres em idade fértil e mortalidade materna tardia de 2010-2017. Os resultados demonstram que a não identificação adequada das mortes maternas tardias reflete a persistente invisibilidade desse grave e importante evento, tanto do ponto de vista social quanto do sanitário ^15^.

Diante da escassez de estudos sobre mortalidade materna tardia, cujas informações são fundamentais para embasar políticas públicas, ações e estratégias de enfrentamento, a temática desperta preocupações e questionamentos pela extensão do problema ^14^ ^,^ ^16^. O objetivo deste estudo foi analisar a tendência temporal da RMMT no Brasil e suas regiões geográficas no período de 2010 a 2019, utilizando dados provenientes do Sistema de Informação sobre Mortalidade (SIM) e do Sistema de Informação sobre Nascidos Vivos (SINASC) do Ministério da Saúde.

## Metodologia

Trata-se de estudo com delineamento ecológico exploratório de série temporal, utilizando dados de mortalidade materna tardia do Brasil e regiões geográficas, no intervalo de 2010 a 2019. Os dados são provenientes do SIM e do SINASC, disponíveis no Portal da Saúde do Departamento de Informática do Sistema Único de Saúde (DATASUS) do Ministério da Saúde. Esses sistemas fornecem a base para a geração de indicadores epidemiológicos, funcionando como matriz estratégica, e podem embasar a tomada de decisão em diversas áreas da atenção à saúde ^17^.

Os dados foram acessados por meio do DATASUS (https://datasus.saude.gov.br/). As informações contidas nas Declarações de Óbito (DO) foram obtidas no diretório “Serviços” (seção de Transferência/Download de Arquivos do SIM/DATASUS/MS). Os dados sobre nascidos vivos foram coletados no diretório “Informações de Saúde” (TABNET), na área de Estatísticas Vitais, opção Nascidos Vivos - 1994 a 2019. Para a utilização dos dados sobre raça/cor materna dos nascidos vivos, realizou-se a importação dos arquivos disponíveis no diretório “Serviços”, (seção Transferência/Download de Arquivos do Sinasc/DATASUS/MS).

Para a leitura/conversão dos arquivos foi utilizado o aplicativo de análise estatística TabWin (http://siab.datasus.gov.br/DATASUS/index.php?area=060805&item=3), uma ferramenta de acesso livre desenvolvida pelo DATASUS. Os bancos de dados foram organizados em planilhas do Microsoft Excel (https://products.office.com/) e posteriormente filtrados para análise dos casos selecionados com a causa básica de morte registrada com o código O96 da Classificação Internacional de Doenças, 10ª revisão (CID-10), indicando óbito materno tardio.

Dessa forma, a população deste estudo foi constituída por todos os óbitos identificados como mortalidade materna tardia na DO, registrados no SIM, de residentes no território brasileiro, no período de 1º de janeiro de 2010 a 31 de dezembro de 2019. A escolha desse período de investigação se deve às modificações nas versões anteriores da DO, visando garantir maior fidedignidade nas informações.

Quanto aos dados descritivos, a análise e a apresentação de tabelas e gráficos foram realizadas utilizando-se o software Microsoft Excel. As variáveis foram expressas em frequência absoluta e relativa. O cálculo da RMMT foi realizado utilizando a fórmula [(número de óbitos maternos tardios/número de nascidos vivos no mesmo período) x 100.000]. Ainda que importante para reduzir imprecisões nos registros, o que pode levar à subnotificação das mortes maternas ^18^, não foi aplicado fator de correção nos valores observados, devido à inexistência de propostas de fatores nacionais de correção indicados para mortalidade materna tardia ^19^.

O Ministério da Saúde orienta o uso de fator de correção de óbitos maternos para fins de cálculo da RMM quando o incremento no número de óbitos maternos no SIM após o processo de vigilância de óbitos for menor que 34,3% (padrão ouro). Entretanto, não há referência sobre validação do uso de fator de correção para óbitos maternos tardios ^19^.

A categorização das variáveis sociodemográficas e demais fatores associados à mortalidade materna tardia seguiu a estrutura do SIM ^17^. No caso das variáveis faixa etária e raça/cor adotou-se a categorização de grupos de idade utilizados pelo Instituto Brasileiro de Geografia e Estatística (IBGE) no *Censo Demográfico 2022* ^20^.

Para a análise das tendências, foi utilizado o pacote estatístico Statistical Software for Professional (Stata, https://www.stata.com), versão 16.0. Considerando a natureza não-estacionária da série temporal de mortalidade materna, foram empregados modelos autorregressivos de Prais-Winsten, nos quais as variáveis dependentes foram as razões de mortalidade materna tardia para o Brasil e suas cinco regiões geográficas.

Adotou-se o modelo de regressão de Prais-Winsten por ser indicado para corrigir a autocorrelação serial proveniente de séries temporais. Para a realização da regressão, as RMMT foram transformadas para a escala logarítmica. Esse processo é realizado visando reduzir a heterogeneidade da variância dos resíduos provenientes da análise de regressão de séries temporais ^18^ ^,^ ^19^.

Além disso, foi calculada a variação percentual média anual (APC) para cada variável dependente analisada. A fórmula utilizada para o cálculo da APC foi a seguinte: APC = (-1+10[b1]*100%), em que b1 representa o coeficiente angular (beta) da regressão de Prais-Winsten ^18^ ^,^ ^19^. Os intervalos de 95% de confiança (IC95%) das medidas de APC foram calculados conforme as fórmulas IC95% mínimo (-1+10[b1-te]*100%) e IC95% máximo (-1+10[b1+te]*100%).

Os valores dos coeficientes angulares (b1) da regressão de Prais-Winsten e erros padrão foram gerados pelo programa de análise estatística; foi realizado o teste t de Student, com 9 graus de liberdade (t = 2,262 para o período de 10 anos), e para a variável raça/cor materna utilizou-se 7 graus de liberdade (t = 2,365 para o período de 8 anos), ambos com nível de 95% de confiança.

Os resultados da regressão foram interpretados da seguinte forma: tendência crescente quando o valor de p foi menor que 0,05 e o coeficiente de regressão foi positivo; tendência decrescente quando o valor de p foi menor que 0,05 e coeficiente de regressão foi negativo; ou tendência estacionária quando o valor de p foi maior que 0,05 ^21^.

Este estudo utilizou dados públicos, disponíveis para pesquisadores e cidadãos, sem restrições. Tais informações foram obtidas de fontes que os apresentaram de forma agregada, garantindo o anonimato das mulheres. Portanto, de acordo com a *Resolução nº 510/2016* do Conselho Nacional de Saúde, não foi necessário submeter o estudo a apreciação por um Comitê de Ética em Pesquisa em Seres Humanos.

## Resultados

No período compreendido entre 2010 e 2019, foram notificados 1.470 óbitos maternos tardios no Brasil, com uma média anual de 147 óbitos (DP = 113-181). No ano de 2018 foi registrado o maior número de óbitos, totalizando 209 casos, correspondendo a 14,2% de todos os óbitos maternos tardios no período. Por outro lado, no ano de 2010 ocorreu o menor número de registros, com 63 óbitos maternos tardios declarados, equivalente a 4,3% do total de óbitos.

Os óbitos maternos tardios no Brasil predominaram entre as mulheres com mais de 45 anos, aquelas com baixa escolaridade e as registradas como viúvas. Em relação à raça/cor, 59,1% (n = 869) ocorreram em mulheres negras ao longo dos dez anos investigados, com uma RMMT de 5,7 por 100 mil nascidos vivos, igual valor da RMMT de mulheres brancas, que representaram 36,9% (n = 542) do total dos óbitos.

Dos óbitos maternos tardios declarados no Brasil durante o período de estudo, 1.230 casos foram investigados, 83,7% do total. Em 131 casos não houve investigação (8,9%), enquanto em 109 casos (7,4%) não houve resposta para essa variável. Entre as mortes investigadas, 65,6% dos casos de mortalidade materna tardia não tinham o registro da causa básica com o código para óbito materno tardio na declaração de óbito original, sendo corrigidos estes dados após investigação pelos Comitês de Mortalidade Materna.

A Região Sudeste destacou-se pelo maior percentual de investigação dos casos de mortalidade materna tardia, com 92,5% (n = 483) dos óbitos maternos tardios investigados entre 2010 e 2019. Os menores percentuais de investigação foram observados na Região Nordeste com 53,3% (n = 218) seguido pela Região Sul com 77,3% (n = 249) e as regiões Centro-oeste e Norte com cobertura semelhante, 84,1% (n = 90) e 84,5% (n = 93), respectivamente.

Ao final dos dez anos investigados, a Região Sudeste apresentou o maior número de casos de mortalidade materna tardia, com 522 óbitos registrados (35,5%), enquanto a Região Centro-oeste apresentou o menor número, declarando 107 óbitos no mesmo período (7,3%). Destaca-se que a Região Norte não declarou óbitos maternos tardios nos anos de 2010 e 2011.

A avaliação das Unidades Federativas (UF) revelou que o Rio de Janeiro registrou o maior número de óbitos, 280 (19%), seguido pelo Rio Grande do Sul, com 213 óbitos registrados (14,5%). O Distrito Federal não declarou nenhum óbito materno tardio ao longo da década, enquanto o Acre e o Amapá registraram apenas um óbito no período. A distribuição dos óbitos maternos tardios por UF/Regiões geográficas brasileiras entre os anos de 2010 e 2019 está apresentada na Tabela 1.

**Tabela 1:** Óbitos maternos tardios. Brasil, regiões e Unidades Federativas (UF), 2010-2019.

Região geográfica/UF	2010-2011		2012-2013		2014-2015		2016-2017		2018-2019		2010-2019	
	n	%	n	%	n	%	n	%	n	%	n	%
Região Centro-oeste	8	4,5	13	5,4	14	3,9	36	11,0	36	9,8	107	7,3
Distrito Federal	-	-	-	-	-	-	-	-	-	-	-	-
Goiás	-	-	1	-	-	-	5	-	11	-	17	1,2
Mato Grosso	2	-	2	-	7	-	17	-	20	-	48	3,3
Mato Grosso do Sul	6	-	10	-	7	-	14	-	5	-	42	2,9
Região Nordeste	47	26,6	56	23,2	91	25,5	77	23,6	138	37,4	409	27,8
Alagoas	4	-	-	-	-	-	1	-	-	-	5	0,3
Bahia	6	-	21	-	20	-	19	-	13	-	79	5,4
Ceará	9	-	8	-	19	-	6	-	57	-	99	6,7
Maranhão	9	-	6	-	7	-	2	-	1	-	25	1,7
Paraíba	-	-	7	-	8	-	10	-	17	-	42	2,9
Pernambuco	1	-	-	-	18	-	22	-	30	-	71	4,8
Piauí	16	-	11	-	16	-	13	-	15	-	71	4,8
Rio Grande do Norte	1	-	-	-	-	-	1	-	4	-	6	0,4
Sergipe	1	-	3	-	3	-	3	-	1	-	11	0,7
Região Norte	-	-	14	5,8	23	6,4	35	10,7	38	10,3	110	7,5
Acre	-	-	1	-	-	-	-	-	-	-	1	0,1
Amapá	-	-	-	-	1	-	-	-	-	-	1	0,1
Amazonas	-	-	3	-	4	-	12	-	19	-	38	2,6
Pará	-	-	5	-	10	-	11	-	13	-	39	2,6
Rondônia	-	-	4	-	6	-	6	-	4	-	20	1,4
Roraima	-	-	-	-	-	-	1	-	1	-	2	0,1
Tocantins	-	-	1	-	2	-	5	-	1	-	9	0,6
Região Sudeste	65	36,7	94	39,0	136	38,1	118	36,2	109	29,5	522	35,5
Espírito Santo	5	-	14	-	29	-	21	-	13	-	82	5,6
Minas Gerais	1	-	4	-	13	-	10	-	7	-	35	2,4
Rio de Janeiro	46	-	52	-	54	-	60	-	68	-	280	19
São Paulo	13	-	24	-	40	-	27	-	21	-	125	8,5
Região Sul	57	32,2	64	26,6	93	26,1	60	18,4	48	13,0	322	21,9
Paraná	19	-	12	-	29	-	18	-	26	-	104	7,1
Rio Grande do Sul	38	-	52	-	62	-	41	-	20	-	213	14,5
Santa Catarina	-	-	-	-	2	-	1	-	2	-	5	0,3
Brasil	177	100,0	241	100,0	357	100,0	326	100,0	369	100,0	1.470	100,0

Fonte: elaboração própria com dados do Departamento de Informática do SUS ^17^.

Quanto à distribuição dos óbitos maternos tardios por município no período de estudo, destaca-se a cidade do Rio de Janeiro, que registrou 175 casos, correspondendo a 11,9% de todos os óbitos declarados no país. Em Porto Alegre (Rio Grande do Sul), segundo município com o maior número de casos, foram declarados 45 óbitos maternos tardios (3,1%) nos dez anos de investigação.

Na análise da mortalidade materna tardia nas cinco regiões geográficas do país em todo o período estudado, a Região Norte apresentou a menor RMMT, de 3,5 por 100 mil nascidos vivos e a mais elevada foi encontrada na Região Sul, 8,3 por 100 mil nascidos vivos, seguida pela Região Nordeste, onde a RMMT foi de 4,9/100 mil nascidos vivos. Ao final dos dez anos, as regiões Centro-oeste e Sudeste apresentaram RMMT iguais, com 4,5 por 100 mil nascidos vivos.

O comportamento da RMMT para o Brasil e regiões ao longo dos anos estudados é expresso na Figura 1. No início do período a RMMT para o Brasil foi de 2,2 óbitos por 100 mil nascidos vivos, evoluindo para 5,6 óbitos por 100 mil nascidos vivos em 2019. Na década estudada, a RMMT no Brasil foi de 5 óbitos a cada 100 mil nascidos vivos. Observou-se variação da RMMT nas cinco regiões geográficas do país: no triênio inicial (2010-2012) as RMMT mais baixas foram verificadas nas regiões Centro-oeste, Nordeste e Norte, com aumento progressivo, ultrapassando as demais regiões no final do período estudado.

**Figura 1:** Razão de mortalidade materna tardia. Brasil e regiões geográficas, 2010-2019.

A análise dos coeficientes da regressão de Prais-Winsten para o Brasil e para cada uma das cinco regiões geográficas demonstrou tendência crescente significativa da RMMT no Brasil (p = 0,003) e nas regiões Centro-oeste, Nordeste e Norte (p < 0,001 nas três regiões), e estacionária nas regiões Sul e Sudeste.

As variações percentuais médias anuais do Brasil e das cinco regiões geográficas mostraram um aumento da RMMT no Brasil, com variação percentual média anual de 9,79% (IC95%: 4,32; 15,54)(Tabela 2). Na análise segundo região, o Centro-oeste apresentou o maior aumento na RMMT, com variação percentual média anual de 26,06% (IC95%: 16,36; 36,56), seguida pela Região Norte com 23,5% (IC95%: 13,93; 33,88) e Região Nordeste, com 14,6% (IC95%: 8,50; 21,05). As demais regiões apresentaram tendência estacionária.

**Tabela 2:** Variação percentual média da razão de mortalidade materna tardia. Brasil e regiões, 2010-2019.

	% variação média anual	IC95%	Valor de p	Tendência
Brasil	9,79	4,32; 15,54	0,003	Crescente
Regiões geográficas				
Centro-oeste	26,06	16,36; 36,56	< 0,001	Crescente
Nordeste	14,60	8,50; 21,05	< 0,001	Crescente
Norte	23,50	13,93; 33,88	< 0,001	Crescente
Sudeste	6,74	-1,19; 15,30	0,092	Estacionária
Sul	-3,26	-11,20; 5,39	0,407	Estacionária

IC95%: intervalo e 95% de confiança.

Fonte: elaboração própria com dados do Departamento de Informática do SUS ^17^.

No final do período estudado constatou-se que 77,3% das mulheres que morreram no puerpério tardio (n = 1.137) receberam atendimento médico durante a enfermidade que ocasionou o óbito. A análise por quinquênios revelou aumento na cobertura de assistência médica, sendo de 70% (n = 417) entre 2010-2014 e 82,4% (n = 720) entre 2015-2019. Destaca-se o expressivo percentual de casos em que essa informação não estava disponível, notadamente entre os anos de 2010 e 2014, quando em 22,3% das ocorrências (n = 133) esse dado não foi registrado. Contudo, a qualidade da informação melhorou no segundo quinquênio, com 9,6% das ocorrências (n = 84) sem registro do dado.

## Discussão

Os achados deste estudo evidenciam uma tendência crescente da RMMT no Brasil e na maioria de suas regiões ao longo da década de 2010-2019, com predomínio das mortes entre mulheres acima de 45 anos, negras, com baixa escolaridade e viúvas. Esses dados indicam as lacunas históricas da injustiça social e reprodutiva que marca a vida das mulheres ^5^ ^,^ ^22^. Além disso, observaram-se falhas importantes na notificação e incompletude de informações na DO original.

Pesquisas anteriores, que investigaram os óbitos na região das Américas de 1999-2013, como o estudo conduzido por Cosio et al. ^14^, sugerem que a tendência ascendente na mortalidade materna tardia pode ser verificada no Brasil desde, pelo menos, a década de 1990. Carvalho et al. ^15^ destacam que, em países desenvolvidos, o aumento das mortalidade materna tardia está associado à ampliação da sobrevida, mas também pode ser atribuído à ampliação da investigação dos óbitos de mulheres em idade fértil ao longo dos anos com a correção do sub-registro, à busca ativa e qualificação e uso de sistemas de informações.

Destaca-se que no Brasil a regulamentação da investigação da mortalidade materna pelos municípios, em todo o território nacional, foi estabelecida pela *Portaria nº 1.119* do Ministério da Saúde apenas em 2008 ^3^. A organização das vigilâncias municipais para esse fim, embora já houvesse iniciativas anteriores com a implantação de Comitês de Mortalidade Materna em alguns estados desde a década de 1990, só se deu a partir de 2009, o que explica em parte o aumento da identificação e notificação de óbitos maternos nos anos que se seguiram.

Embora globalmente tenha ocorrido uma diminuição das mortalidade materna tardia, passando de 8.460 (IC95%: 5.792; 11.935) em 1990 para 6.711 (IC95%: 4.335; 9.996) em 2015 ^8^, vários estudos e relatórios internacionais ^8^ ^,^ ^14^ ^,^ ^23^ ^,^ ^24^ ^,^ ^25^ ^,^ ^26^ ^,^ ^27^ ^,^ ^28^ ^,^ ^29^ ^,^ ^30^ indicam que a mortalidade materna tardia não é um desafio apenas para o Brasil, atingindo países de diversos graus de desenvolvimento. Nas Américas, entre 1999 e 2013 foi observada tendência crescente na mortalidade materna tardia, passando de 1.179 óbitos entre 1999 e 2006 para 3.153 entre 2006 e 2013, com variação percentual média anual de 12,4, sendo a maior diferença observada nos Estados Unidos (15,4%), seguido pelo México (15,1%) ^14^. A RMMT e a RMM por sequelas de causas obstétricas foi duas vezes maior na região das Américas entre 2006 e 2013, comparado com o período de 1999 a 2005.

Neste estudo, verificou-se a RMMT de cinco óbitos por 100 mil nascidos vivos entre 2010 e 2019 para o Brasil. Relatórios oficiais indicam RMMT de 13,7 óbitos por 100 mil nascidos vivos entre 2018 e 2020 no Reino Unido ^25^ e de 2,7 óbitos por 100 mil nascidos vivos entre 2013 e 2015 na França ^24^. Parte da discrepância das taxas entre os países se deve a diferenças no registro e na qualidade das informações.

No Brasil, embora o Ministério da Saúde tenha buscado desenvolver estimativas de RMM aplicáveis com confiança no nível subnacional, considerando as desigualdades regionais, essas estimativas não incluem a mortalidade materna tardia devido à baixa completude e confiabilidade na investigação dessas mortes. Soma-se a isso a escassa produção de conhecimento sobre o tema. As limitações na identificação e registro de mortalidade materna tardia resultam da distância temporal entre a ocorrência do óbito e o parto, dificultando a definição do nexo causal entre causa do óbito e condições da gestação, parto e/ou puerpério ^31^.

Essa dificuldade também pode estar relacionada à subinformação ou subnotificação desses óbitos, o que demanda revisão e maior detalhamento dos códigos para as mortes materna tardia, bem como ampla capacitação dos profissionais de saúde, especialmente de médicos, responsáveis pelo preenchimento da DO, assim como de técnicos codificadores de óbitos no Brasil e dos profissionais que compõem os comitês de vigilância do óbito materno ^13^ ^,^ ^31^. A subnotificação ocorre também em países desenvolvidos ^8^ ^,^ ^23^ ^,^ ^24^.

Além disso, há dificuldade na atribuição correta dos códigos da CID-10 para óbitos maternos tardios e não-tardios no processo de codificação das declarações de óbito. Esse desafio afeta principalmente áreas de pior nível socioeconômico, nas quais a mortalidade materna tende a ser mais alta ^31^ ^,^ ^32^.

A utilização dos códigos referentes às mortes maternas tardias (O96) na CID, incluída pela OMS na revisão da CID-10 em 2015 (códigos O96.0 - óbito materno tardio obstétrico direto; O96.1 - óbito materno obstétrico indireto e O96.9 - para causas indefinidas), deve ser implementada, considerando a magnitude da mortalidade materna tardia, com o treinamento das equipes de vigilância epidemiológica, os comitês e os codificadores, com vistas a aprimorar a vigilância, a investigação, a codificação e a análise dos óbitos maternos tardios. A adoção dessa codificação no Brasil oferecerá maior fidedignidade e, consequentemente, visibilidade aos óbitos maternos tardios ^1^.

Autores destacam a complexidade de se mensurar com precisão a mortalidade materna tardia devido à subnotificação e os desafios no diagnóstico das causas subjacentes de óbito ^13^. Embora seja obrigatória a notificação da morte materna, o sub-registro prejudica o real dimensionamento desses óbitos ^4^ ^,^ ^8^ ^,^ ^13^ ^,^ ^16^ ^,^ ^33^. No Brasil verificaram-se avanços consideráveis nos registros oficiais de óbitos, e as investigações, apesar de abrangerem uma cobertura superior a 90%, não são uniformes em todo o país, variando de 53,3% no Nordeste a 92,3% no Sudeste.

Os comitês de mortalidade materna têm papel decisivo na identificação e correção da subnotificação de óbitos maternos, especialmente os tardios, cujas causas são complexas para identificação e análises. Vega et al. ^13^ enfatizam que sem a atuação dos comitês, as mortes maternas tardias permaneceriam ocultas, dada sua maior subnotificação em relação às mortes em até 42 dias do puerpério.

É importante destacar que o cenário da mortalidade materna tardia no Brasil diverge daquele da RMM (até 42 dias). Enquanto a RMMT apresenta tendência crescente de 2010-2019, a tendência da RMM é de estagnação, com leve queda até o período de pandemia, quando houve brusca elevação, quase dobrando a RMM no país ^34^. Ambos os indicadores apresentam disparidades regionais, indicando que, no período do estudo, houve redução significativa da RMM nas regiões Centro-oeste, Nordeste e Sul ^35^ Em contraponto, foi evidenciada a tendência crescente da RMMT na região Centro-oeste, Nordeste e Norte, enquanto nas regiões Sudeste e Sul foi identificada uma tendência de estabilidade.

Essa tendência estacionária da RMM, pode ser, em parte, explicada pelo chamado paradoxo perinatal, o qual destaca a persistência de taxas elevadas de mortalidade materna, apesar dos avanços tecnológicos e recursos disponíveis. Isso é atribuído à hipermedicalização do processo de parto e nascimento, caracterizada pelo excesso de intervenções que impactam o curso fisiológico natural, resultando no aumento do risco de complicações e mortalidade ^36^.

A crescente RMMT no país pode sugerir uma melhoria na qualidade do registro da informação dessas mortes, com aumento e qualificação da investigação do óbito materno no decorrer do período. Por outro lado, pode indicar a desigualdade na sobrevida materna devido a problemas e disparidades no acesso, disponibilidade e qualidade dos serviços de saúde, infraestrutura, educação e condições socioeconômicas, como o acesso ao cuidado pré-natal, ao parto e acompanhamento pós-parto seguros em diferentes regiões do Brasil ^34^. É fundamental compreender melhor as diferenças regionais, para orientar a elaboração de políticas públicas de saúde no país de acordo com necessidades locais.

O reconhecimento da importância da RMMT e sua inclusão nos indicadores de saúde nos compromissos governamentais e no planejamento em saúde é essencial para a compreensão e abordagem efetiva e prevenção das mortes maternas. Embora a Agenda 2030 dos ODS tenha como meta apenas a redução da mortalidade materna até 42 dias de puerpério, seria recomendável a inclusão da redução da mortalidade materna tardia em futuras metas e objetivos de desenvolvimento do Brasil e todos os países, considerando as evidências de sua magnitude, transcendência e evitabilidade ^4^ ^,^ ^14^ ^,^ ^15^. Adicionalmente, as estatísticas oficiais mais recentes do Ministério da Saúde evidenciam que o Brasil está consideravelmente distante de cumprir as atuais metas dos ODS ^35^.

Este estudo apresenta limitações, entre as quais a utilização de dados secundários de registros de óbitos e da elevada proporção de subnotificação. Os resultados, portanto, devem ser entendidos como restritos ao subconjunto dos óbitos registrados no SIM, tendo em vista a possibilidade de viés de informação, sobretudo em regiões onde é mais alto o sub-registro de mortes maternas. Entretanto, as análises realizadas neste estudo mostram pela primeira vez um panorama da mortalidade materna tardia no Brasil durante uma década, 2010-2019, com potencial para fomentar discussões e nortear avanços das ações em saúde que alcancem todas as mulheres. A invisibilidade das mortes maternas tardias, evidenciada pela sua subnotificação nos sistemas de informação oficiais, e revelada também neste estudo, constitui mais um aspecto da injustiça reprodutiva do nosso país, aprofundando a lacuna e o atraso do Brasil em relação à saúde, a garantia de direitos reprodutivos e de vida digna para as mulheres.

Destaca-se, em particular, a importância de se dedicar atenção especial ao acompanhamento das mulheres durante o puerpério tardio, considerando o potencial impacto positivo na prevenção dessas mortes evitáveis. Recomenda-se, portanto, que futuras pesquisas incorporem uma análise aprofundada sobre as causas subjacentes da mortalidade materna tardia, além de intervenções na formação profissional e na melhoria da qualidade dos serviços de saúde.

Essas e outras medidas podem contribuir para o alcance do compromisso de redução da mortalidade materna e promoção da saúde das mulheres, destacando-se os desafios associados à assistência e cuidado com as mulheres no puerpério.
